# Crosstalk Between Autophagy and Innate Immunity: A Pivotal Role in Hepatic Fibrosis

**DOI:** 10.3389/fphar.2022.891069

**Published:** 2022-05-17

**Authors:** Li Chen, Desong Kong, Siwei Xia, Feixia Wang, Zhanghao Li, Feng Zhang, Shizhong Zheng

**Affiliations:** ^1^ Jiangsu Key Laboratory for Pharmacology and Safety Evaluation of Chinese Materia Medica, School of Pharmacy, Nanjing University of Chinese Medicine, Nanjing, China; ^2^ Chinese Medicine Modernization and Big Data Research Center, Nanjing Hospital of Chinese Medicine Affiliated to Nanjing University of Chinese Medicine, Nanjing University of Chinese Medicine, Nanjing, China

**Keywords:** liver fibrosis, autophagy, innate immunity, HSCs, immune cells

## Abstract

Liver fibrosis is a repair process of chronic liver injuries induced by toxic substances, pathogens, and inflammation, which exhibits a feature such as deposition of the extracellular matrix. The initiation and progression of liver fibrosis heavily relies on excessive activation of hepatic stellate cells (HSCs). The activated HSCs express different kinds of chemokine receptors to further promote matrix remodulation. The long-term progression of liver fibrosis will contribute to dysfunction of the liver and ultimately cause hepatocellular carcinoma. The liver also has abundant innate immune cells, including DCs, NK cells, NKT cells, neutrophils, and Kupffer cells, which conduct complicated functions to activation and expansion of HSCs and liver fibrosis. Autophagy is one specific type of cell death, by which the aberrantly expressed protein and damaged organelles are transferred to lysosomes for further degradation, playing a crucial role in cellular homeostasis. Autophagy is also important to innate immune cells in various aspects. The previous studies have shown that dysfunction of autophagy in hepatic immune cells can result in the initiation and progression of inflammation in the liver, directly or indirectly causing activation of HSCs, which ultimately accelerate liver fibrosis. Given the crosstalk between innate immune cells, autophagy, and fibrosis progression is complicated, and the therapeutic options for liver fibrosis are quite limited, the exploration is essential. Herein, we review the previous studies about the influence of autophagy and innate immunity on liver fibrosis and the molecular mechanism to provide novel insight into the prevention and treatment of liver fibrosis.

## 1 Introduction

### 1.1 Overview, Pathology, and Pathogenesis of Liver Fibrosis

Liver fibrosis is a chronic disease caused by liver injuries that are stimulated by several factors, such as excessive alcohol consumption, virus infection (including hepatitis B and hepatitis C), non-alcohol steatohepatitis (NASH), non-alcoholic fatty liver disease (NAFLD), and autoimmune hepatitis (Affo et al., 2017; Kakino et al., 2018; Parola and Pinzani 2019). As a result of multiple kinds of liver damage and diseases, liver fibrosis has a large prevalence and high mortality in China and also worldwide (Moon et al., 2020). Liver fibrosis will gradually result in hepatocellular carcinoma without timely treatment (Affo et al., 2017). The features of liver fibrosis are excessive accumulation of extracellular matrix (ECM), which consists of glycoproteins, collagen, and proteoglycans (Karsdal et al., 2017). Hepatic stellate cells (HSCs) are recognized as the main cause for initiation and progression of liver fibrosis (Li X. et al., 2020). The hepatic damage causes the activation of HSCs, which are changed from the quiescent status to myofibroblasts, and leads to production and accumulation of the ECM, ultimately causing liver fibrosis (Zhang et al., 2016; Chen et al., 2019; Dewidar et al., 2019). The progression of liver fibrosis will lead to mild-to-moderate liver fibrosis, and nearly 30% of the patients will develop cirrhosis, in which more than 80% of patients will finally progress to hepatocellular carcinoma (Pang et al., 2018). Notably, liver fibrosis is a reversible pathological condition, lacking effective treatment in addition to surgical resection or transplantation (Baglieri et al., 2019). However, if the disease developed to terminal stage, the surgical operation will be invalid. As a result, it is significant to prevent the initiation and progression of liver fibrosis.

There are different factors involved in the activation of HSCs, including toxins, hepatitis, autoimmune disorders, and steatohepatitis. HSC activation contains two major stages, which are named as initiation and perpetuation, to stimulate the HSCs to acquire a myofibroblast-like phenotype (Higashi et al., 2017). Early activation of HSCs is driven by paracrine and autocrine growth factors, mainly including platelet-derived growth factor (PDGF), fibroblast growth factor (FGF), vascular endothelial growth factor (VEGF), and transforming growth factor (TGF-β) (Borkham-Kamphorst and Weiskirchen 2016; Xu et al., 2016; Feng et al., 2019; Schumacher et al., 2020). The release of these growth factors is regulated at the transcriptional level. Moreover, the continuous stimuli for HSC activation will result in alteration of cell behaviors, such as proliferation, fibrogenesis, matrix degradation, and cytokine release, finally causing continuous activation (Zhou et al., 2019). Overall, these cellular changes will contribute to ECM accumulation. The ECM mainly comprises *α* smooth muscle actin (α-SMA) and collagen, and the excessive accumulation of the ECM will cause damage to normal liver structure and function (Schnieder et al., 2020).

The role of autophagy in the process of liver fibrosis is complex and bidirectional, which has received particular attention in recent years. From the current research, the upregulation of autophagy can aggravate liver fibrosis mainly by promoting HSC activation in HSC autophagy, especially lipophagy can provide energy for its activation (Hou et al., 2022). The inhibitory effect of upregulated autophagy on liver fibrosis is mainly reflected in the protective effect of hepatocyte and LSEC autophagy and the anti-inflammatory effect of immune cell autophagy (Hammoutene et al., 2020). In addition, various signaling pathway studies suggest that the upregulation of HSC autophagy aggravates liver fibrosis (Liu et al., 2020). Autophagy of different kinds of cells and different levels of autophagy of different substrates may have biased or different mechanisms on liver fibrosis (Li Y. et al., 2020). In addition, its complexity may be related to the type of disease and period of fibrosis.

### 1.2 Immune Cells, Inflammation, and Liver Fibrosis

In the traditional conception, the liver is a metabolic organ conducting the function of substance circulation and energy regeneration. However, the unique anatomical characteristics of the liver, including abundant blood supply and rich microarchitecture of vessels, have empowered the liver with a profound feature of immune function (Jenne and Kubes 2013). The liver is abundant in multiple immune cells, which contribute to immune regulation of hepatic function. Compared with adaptive immunity, the innate immune response is activated much faster, especially in acute liver injury, where the host has little time to trigger an effective adaptive immune response. From this perspective, the innate immune system may provide a more profound contribution than the adaptive immune system (Wu et al., 2010). A coordinated network of innate immune cells, inducing Kupffer cells (KCs), natural killer cells (NKs), innate lymphoid cells (ILCs), mucosal-associated invariant T cells (MAITs), dendritic cells (DCs), and invariant NKT cells (iNKTs), are involved in the inflammation of the liver to encounter the invading microorganisms, toxic damage, and pathological challenges (Hossain and Kubes 2019). Typically, KCs are responsible for first detecting the occurrence of exogenous stimulation, releasing the proinflammatory cytokines (such as IL-6, TNF-α, and IL-6), and chemokines (e.g., CCL-2, CCL-4, and CXCL-1) (Abdullah and Knolle 2017). The soluble factors contribute to the recruitment of monocytes and neutrophils to the liver. Neutrophils play an important role in innate immune response by capturing and destroying the pathogens, performing its function by producing reactive oxygen species (ROS), stretching neutrophil extracellular traps (NETs), and conducting the function of phagocytosis (Yang et al., 2019). DCs conduct the functions by bridging the gap between innate immune reaction and adaptive immune response. DCs maintain quiescent conditions under a healthy environment, but they move to the lymph node after stimulation and present antigens to T cells to further activate adaptive immune reaction (Soysa et al., 2017). In addition, the abovementioned cell types and the innate-like lymphocytes, such as MAITs, ILCs, NKs, and iNKTs, are also significant in shaping the hepatic immune microenvironment by secreting different kinds of cytokines (Chen and Tian 2020; Niehaus et al., 2020; Gan et al., 2021). The cytokines and chemokines further facilitate the expression of adhesive molecules such as VCAM1 and ICAM1 to stimulate the secretion of hepatic sinusoidal endothelial cells and ECM formation (Roh and Seki 2018).

**FIGURE 1:**
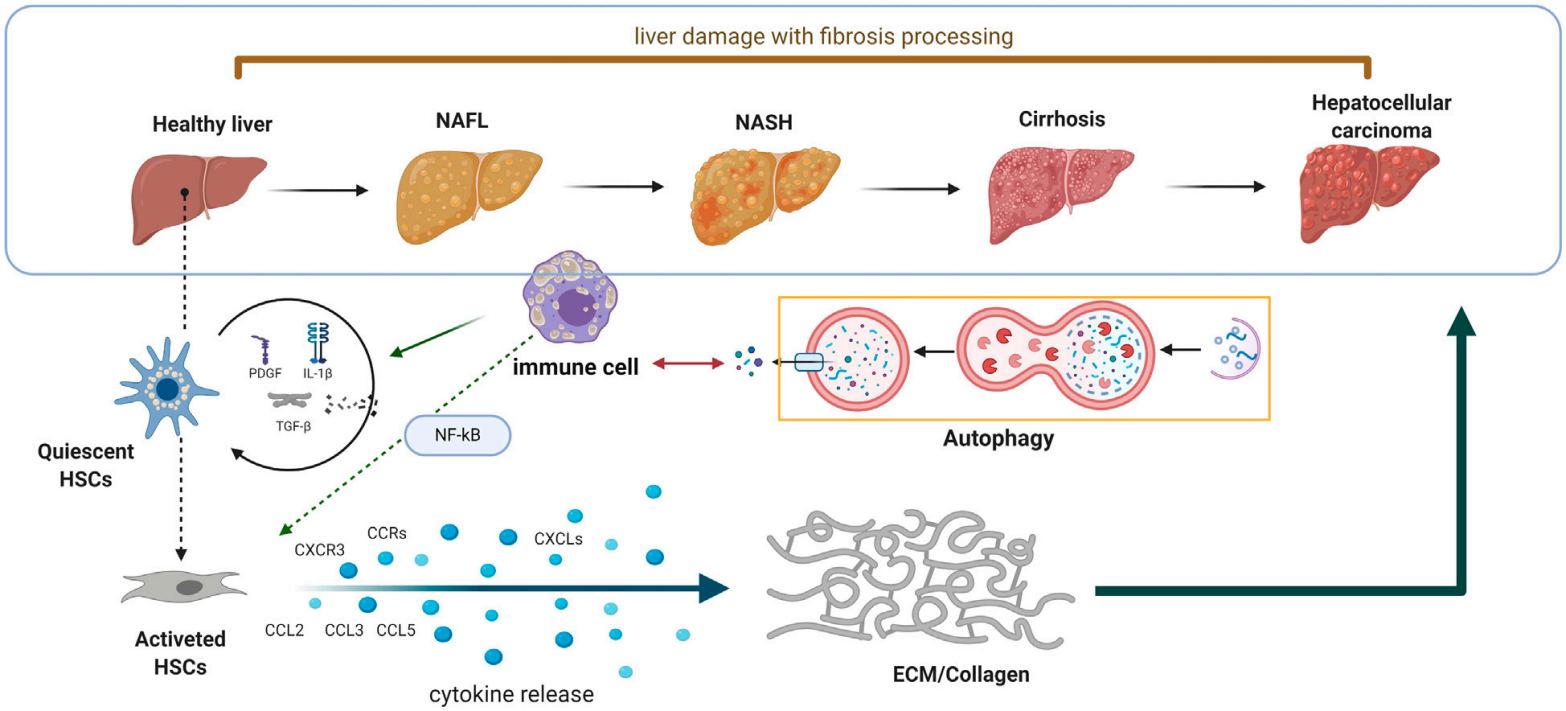
Schematic diagram of the interaction between autophagy and innate immunity in the process of liver fibrosis. When the liver encounters sustained damage, the immune cells will generate huge amount of chemokines and cytokines to exert its defensive reaction. Meanwhile, the HSCs also express different kinds of chemokine receptors to further promote matrix remodulation.

When the liver encounters sustained damage, the inflammatory cells will generate a huge amount of chemokines and cytokines to exert its defensive reaction (Figure 1). Meanwhile, the HSCs also express different kinds of chemokine receptors. The chemokine receptors dysregulated in HSCs contain CXC chemokine receptor 3 (CXCR3); C–C chemokine receptor (CCR) family, including CCR5 and CCR7(Seki et al., 2009). The chemokine family chain in HSCs contains the chemokine ligand (CCL) family, which includes CCL2, CCL3, and CCL5; in addition, the CXC chemokine ligand (CXCL) family contains CXCL1 and CXCL8–CXCL10 (Weiskirchen and Tacke 2014; Yan et al., 2020). Generally, the chemokines bind to their receptors to promote HSC migration and ECM generation. However, the function of chemokines and their receptors in liver fibrosis is a double-edged sword. For instance, the overexpression of CCR1, CCR5, and CXCL4 can contribute to progression of liver fibrosis, whereas the stimulation of CXCR3 and CXCL9 exert the protective function of liver fibrosis (Bruns et al., 2014; Arsent’eva et al., 2015; Ambade et al., 2019).

In addition, to the chemokines and their receptors in the initiation and progression of liver fibrosis, the secreted cytokines also enhance the inflammatory effect. The immune cells and parenchymal cells secrete interleukin-1β, transforming growth factor (TGF-β), tumor necrosis factor-α (TNF-α), and other kinds of cytokines to enhance the inflammatory effect of hepatocytes and activated HSCs (Mridha et al., 2017; Fabre et al., 2018; Kakino et al., 2018). The downstream pathways of these cytokines were also partially elucidated in the previous studies. For instance, the TGF-β/Smad signaling is deeply involved in HSC activation. After activation by TGF-β/Smad signaling, the HSCs will, in turn, generate TGF-β in a manner of autocrine function, which consequently enhances the activation of the TGF-β/Smad pathway. In addition, the NF-κB pathway is also associated with liver fibrosis. The stimuli of the NF-κB pathway include the proinflammatory factors including interleukins (IL-1, IL-2, and IL-6) and TNF-α (Luedde and Schwabe 2011). The signaling of the NF-κB pathway in fibrosis also conducts its function in a manner of autocrine function, for the activation of the NF-κB signal will contribute to the translocation of IκB in the nucleus and upregulate the expression of proinflammatory cytokines, finally leading to cascade amplification of the proinflammatory effect and HSC activation (Bai et al., 2018).

Generally, considering the crucial role of hepatic innate immune regulation, the liver is not only a metabolic organ but also an important immune organ that wipes out invading pathogens and endogenous harmful stimuli.

## 2 The Role of Autophagy in the Liver

Autophagy is one specific type of cell death, by which the aberrantly expressed protein and damaged organelles are transferred to lysosomes for further degradation. The initiation and process of autophagy involve the formation of multiple biofilm structures. The autophagosomes are formed, fusing with lysosomes to form autolysosomes, which consequently conduct the function of degradation (Li Y. et al., 2020; Lorincz and Juhasz 2020).

The autophagy flux is regulated by a series of evolutionarily conserved proteins. For instance, the translation products of ATG genes are responsible for the formation of autophagosomes, which could be divided into two major stages: nucleation and elongation (Li and Zhang 2019). The proteins of Atg1/ULK kinase and phosphatidylinositol 3 (PI3K) kinase and the downstream PI3P effectors are critical to the nucleation stage, and the Atg8 and Atg12 are significantly involved in the elongation stage (Mizushima 2010). In addition, there still exists a homolog of yeast Atg8, microtubule-associated protein 1 light chain 3 (LC3), localizing to the autophagosome membrane after posttranslational modification (Lorincz and Juhasz 2020). The clipping product of the C-terminal fragment of LC3 is called LC3-I, which could be activated by Atg7 and transferred to Atg3, and it combines with phosphatidylethanolamine in a manner of covalent bonding, ultimately converting to LC3-II, whose number is closely related to the number of autophagosomes. Under the condition of an adverse situation or pathological factors, the LC3-I will rapidly transform to LC3-II to enhance the autophagy flux, and the expression of LC3-I and LC3-II is an effective parameter to monitor autophagic activity (Huang and Liu 2015). Moreover, the complex of p62/SQSTM1 directly binds to LC3 and is incorporated into the autophagosome to facilitate the process of autophagy, which will be degraded by autophagy ultimately (Katsuragi et al., 2015).

Autophagy commonly occurs in nearly all cell types and is crucial for sustaining cell homeostasis, cell survival, differentiation, and growth (Hu et al., 2019; Yan et al., 2019). Due to its wide distribution and function in nucleated cells, the maintenance of regular autophagy is essential to cellular function (Xiang et al., 2020). It is widely recognized that dysregulation of autophagy is closely related to the development of various diseases, such as malignant tumors (Mowers et al., 2018; Jiang et al., 2019), auto-immune disease (Cosin-Roger et al., 2017), metabolic disease (Yan et al., 2017; Madhavi et al., 2019), and neurodegeneration (Plaza-Zabala et al., 2017; Liu et al., 2019), including liver fibrosis (Qu et al., 2017; Meng et al., 2018; Liu et al., 2019). Similar to the function of autophagy in other types of diseases, autophagy also exerts complicated roles in liver fibrosis. The role of autophagy in liver fibrosis depends on the cell type and stage of the disease. For example, in hepatocytes, autophagy has been demonstrated to have a protective role (Allaire et al., 2019; Hammoutene et al., 2020). In Atg5 liver-specific KO mice, the number of fibrosis-related genes, such as Col-1, *α*-SMA, and TGF-β, was increased in hepatocytes. While in the wild-type mice, saracatinib markedly suppressed the expression of *α*-SMA in HSCs and decreased the TGF-β–induced CTGF expression by increasing autophagy flux in hepatocytes (Li Y. et al., 2020). In HSCs, the function of autophagy becomes paradoxical. On the one hand, it has been proposed that autophagy in HSCs might induce their activation through lipophagy, a selective type of lipid droplet degradation (Hammoutene et al., 2020). On the other hand, converse findings suggest that increased autophagy in HSCs attenuates liver fibrosis. In a recent study, researchers demonstrated that mammalian target of rapamycin (mTOR), an inhibitor of autophagy, actually promotes HSC activation and liver fibrosis through extracellular vesicle (EV) release, while restoring autophagy in HSCs attenuates liver fibrosis by inhibiting the release of fibrogenic EVs(Gao et al., 2020).

**FIGURE 2:**
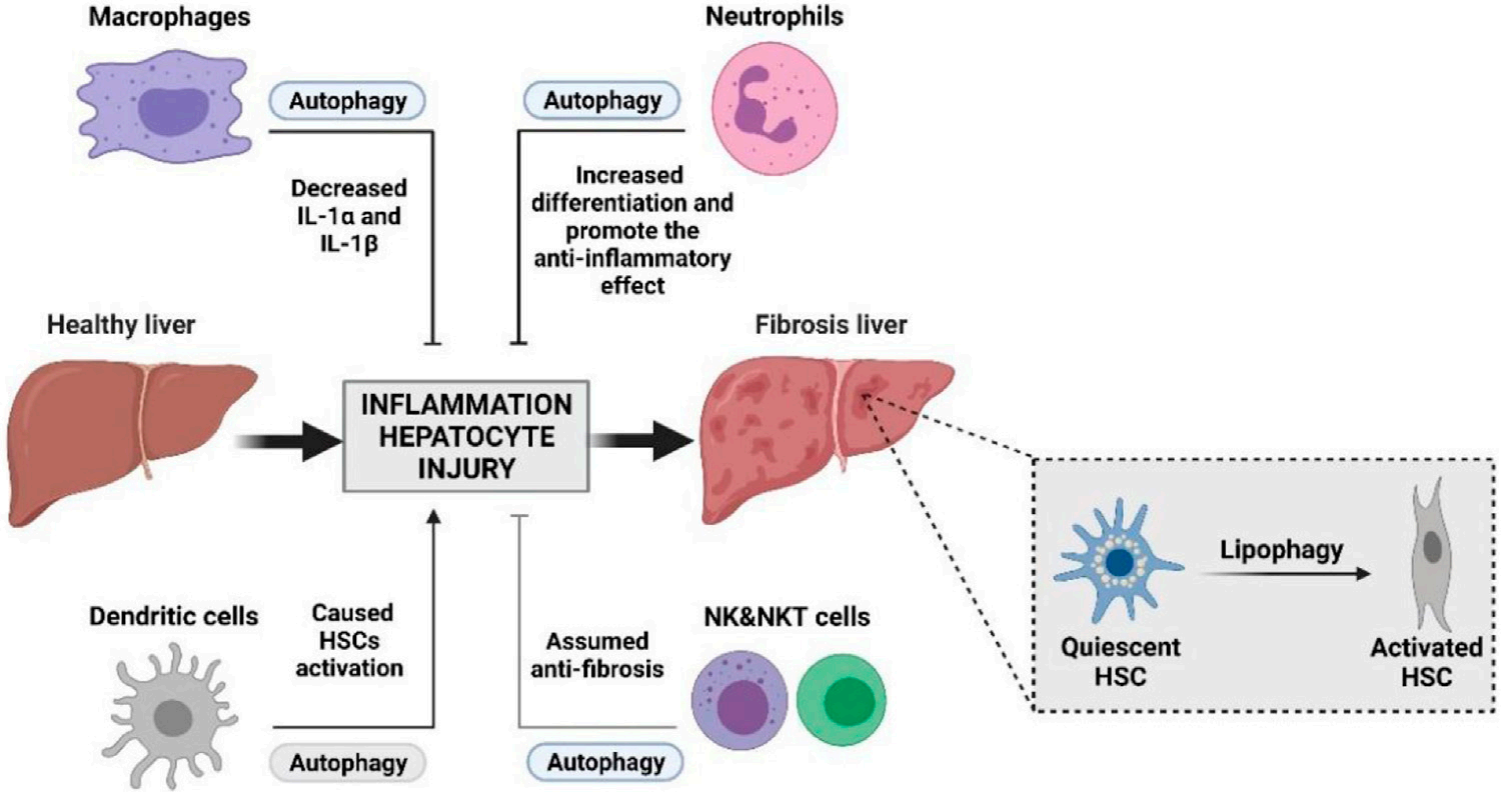
Interplay between autophagy and innate immunity during liver damage with fibrosis processing. Innate immunity plays an important role in liver fibrosis. Activation of HSCs and the remodeling of the hepatic microenvironment by accumulation of the ECM is essential to the initiation and progression of liver fibrosis. The interplay between autophagy and innate immunity may become an important part of intervention in the treatment of liver fibrosis.

As is known, the innate immune cells of the liver are crucial to the influence of liver fibrosis; the stability of liver immune function is an important factor to maintain normal liver function (Wu et al., 2020; Sepulveda-Crespo et al., 2021). Autophagy balances inflammation in innate immunity, and when liver injury occurs, the autophagy function of liver innate immune cells will also change. A failure in autophagy functions is often manifested as dysregulated inflammation in animal models and human diseases (Deretic and Levine 2018). In liver diseases, the functions of immune cells are more complex, and the interaction between autophagy and immune cells may be the underlying molecular mechanism for the complex regulatory functions of immune cells. The formation and progression of fibrosis is a complicated process, which involves the crosstalk between autophagy and innate immune cells (Figure 2). Therefore, a deep understanding of the cellular function of the molecular mechanism of autophagy in liver fibrosis is essential to its therapy.

## 3 Function and Mechanism of Autophagy and Innate Immunity in Liver Fibrosis

The formation and progression of fibrosis is a complicated process, which involves the crosstalk between autophagy and innate immune cells and molecules. Many studies have reported the autophagy function of some major immune cells in liver disease. Here, we summarized the relationship between these major immune cells and autophagy in the process of liver fibrosis as below.

### 3.1 Kupffer Cells

Kupffer cells (KCs) are hepatic macrophages that are a heterogenous population of non-parenchymal cells, comprising 90% of all macrophages, and can be divided into resident and infiltrating macrophages. Kupffer cells are essential for sensing tissue damage and initiating inflammatory response, while infiltrating monocytes/macrophages are associated with chronic inflammatory fiber formation (Ji et al., 2020), (Lodder et al., 2015). Recent studies have shown that during the regression of fibrosis, infiltrating monocytes/macrophages differentiate into “repair” macrophages, which have the function of promoting the regression of fibrosis (Zhu et al., 2018), (Zhou et al., 2018). It is now recognized that KCs play an important role in the pathogenesis of hepatic fibrosis and may be a potential therapeutic target for hepatic fibrosis (Cheng et al., 2021). KCs are involved in the initiation and progression in different stages. In the initiation stage, KCs interact with damage-associated molecular patterns (DAMPs) released by injured or dying hepatocytes, thereby promoting the activation, polarization, and recruitment of KCs. Correspondingly, the activation of KCs induces the secretion of proinflammatory and fibrotic cytokines that act as proinflammatory drivers and promote the activation and fibrotic response of HSCs (Pradere et al., 2013). Current studies have confirmed that HSCs can interact with KCs to promote the generation of the immune microenvironment around HSCs and remodeling of the ECM. The interaction between KCs and HSCs is a complex process involving various cytokines and chemokines (e.g., transforming growth factor (TGF)-β, tumor necrosis factor (TNF)-α, interleukin (IL)-1β, and CCL2) involved in the activation of HSCs. In liver tissue and KCs of animals with CCl_4_-induced fibrosis, the expression of TNF-like ligand 1A was significantly increased, which in turn stimulated the secretion of PDGF-BB, TNF-α, and IL-1β, thereby driving the activation and proliferation of HSCs (Matsuda et al., 2018). In addition, in the period of progression, KCs also release matrix metalloproteinases (MMPs) to remodel the extracellular matrix which further propels liver fibrosis (Cai et al., 2018; Ge et al., 2020). However, some studies have shown that stimulating KCs can also play an antifibrotic role under certain pathological conditions. For example, one study showed that in CCl_4_-induced liver fibrosis, IL-22 polarized M2 KCs through the STAT3 pathway, exerting an anti-inflammatory effect (Su et al., 2021). Another study showed that inhibition of dipeptidyl peptidase 4 (DPP4) skews KCs toward the anti-inflammatory M2 phenotype, thereby alleviating insulin resistance, steatohepatitis, and fibrosis in cholesterol high-fat diet models (Sakai et al., 2020).

In the initiation and progression of liver fibrosis, autophagy in KCs is a potent protective factor for the liver. Autophagy of KCs protects hepatocytes upon liver injury and inhibits hepatic inflammation and fibrogenesis by suppression of IL-1α/β secretion induced by ROS in the mice with CCl_4_ treatment and N-diethylnitrosamine (DEN)–fed rats (Sun et al., 2017). It also limits IL1A and IL1B secretion, thus alleviating the recruitment of other inflammatory cells. While knockout of Atg5, a key player in autophagy, has been proven to aggravate liver fibrosis (Lodder et al., 2015), IL-7 has been confirmed to promote liver injury and inflammation by suppressing the autophagy of KCs (Zhu et al., 2018). IL-7/IL-7R signaling inhibits *Schistosoma japonicum* egg antigen–triggered macrophage autophagy through amp-activated protein kinase (AMPK) signaling, thereby aggravating liver fibrosis in *Schistosoma japonicum* infection (Zhu et al., 2018). Furthermore, spermine can alleviate liver injury by hindering the proinflammatory response of KCs by activating autophagy, which also contributes to the polarization from M2 to M1 (Zhou et al., 2018). Considering the benefits of autophagy, modulating the autophagic function of KCs is an encouraging antifibrotic strategy that deserves further study.

### 3.2 Neutrophils

Neutrophils account for nearly 60% of circulating leukocytes in human bodies, defending the microbial infections in the first line. Neutrophils exert the function of killing pathogens by directly swallowing, producing reactive oxygen species (ROS), and stretching neutrophil extracellular traps (NETs) (Papayannopoulos 2018). In addition to the traditional recognition of the function carried out by neutrophils, emerging evidence also illustrates that neutrophils can interact with other immune cells, including NKs, DCs, and lymphocytes. Neutrophils interact with other immune cells *via* secreting cytokines (such as CCL3 and CCL20) or expressing molecules on the cell membrane, such as peptides.

A systematic review has analyzed the correlation between the neutrophil-to-lymphocyte ratio (NLR) and liver fibrosis, which found that a high NLR was related to mild progression of liver fibrosis in patients infected by HBV, but the patients with chronic HCV infection showed no significant relationships. Generally, the relative number of neutrophils in blood could be associated with the liver fibrosis stage and used as a biomarker in cirrhotic patients (Peng et al., 2018; Pokora Rodak et al., 2018). For mechanism, the previous study found that neutrophils in the liver could induce the proinflammatory effect of macrophages by upregulating miR-223 and downregulating its targeted gene, NLRP3, finally contributing to the spontaneous resolution of liver fibrosis (Calvente et al., 2019).

Accumulative studies have shed light on the role of autophagy in the function of neutrophils, which could further explain the effect of crosstalk between autophagy and neutrophils. During sepsis, the neutrophil autophagy facilitates NET formation and enhances the ability of antibiosis (Park et al., 2017). In a model of ATG16L1-deficient mice, the amount of ROS has increased and the capability of *Salmonella typhimurium* clearance is impaired, which illustrated that autophagy is beneficial to neutrophils in the function of antibiosis. Moreover, autophagy is also important to neutrophil differentiation. Neutrophils are short-lived mediators of innate immunity, which require timely refreshment and constant replenishment. The impairment of autophagy by knocking out ATG7 was proven to enhance glycolytic activity but decreased mitochondrial respiration, reduced ATP production, and enhanced lipid droplets, finally destructing neutrophil differentiation (Riffelmacher et al., 2017). Taken together, we can assume that properly enhanced autophagy in neutrophils will increase their differentiation and promote the anti-inflammatory effect in the liver, which contribute to the remission of liver fibrosis.

### 3.3 Dendritic Cells (DCs)

Dendritic cells (DCs) are heterogenous innate immune cells located in the liver, performing the function of presenting antigens to other immune cells, which are the crucial linkage between innate and adaptive immunity (Théry and Amigorena 2001). The absolute amount of DCs in the liver is not large; thus, hepatic DCs tend to be tolerogenic apart from being immunogenic. After treatment with thioacetamide and leptin, the DCs showed great capability to remodel the proinflammatory environment of liver fibrosis (Izawa et al., 2014). In the condition of liver fibrosis, the number of DCs dramatically increases and obtains an immunogenic phenotype. The previous study has shown that DCs extracted from tissues of liver fibrosis could activate HSCs directly *in vitro* by upregulating the expression of ICAM-1 (Blois et al., 2014). In addition, the overexpression of DCs can activate inflammatory signaling in HSCs and enhance their activation. Thus, the aberrant dynamics of DCs is responsible for the initiation and progression of liver fibrosis, whereas the deterioration of fibrosis increases the population of DCs reciprocally. After being activated by damaged hepatocytes, innate immune cells, especially DCs, recruit and activate CD8^+^T effector cells, which in turn aggravate hepatocyte injury by releasing cytotoxic cytokines (Parola and Pinzani 2019). DCs are critical for the regulation of liver immunity, and irregular DC activity can induce the activation of T cells and HSCs to contribute to the pathological inflammation–rich environment and fibrogenesis (Miao et al., 2015). A recent study on the mechanism of kinsenoside’s antihepatic fibrosis showed that CD8^+^ T cell activation could be blocked by specifically upregulating PD-L1 expression on DCs, while reducing the glycolysis of DCs and keeping DCs in the immature state, improved the liver fibrosis index (Xiang et al., 2022).

As a mediator to deliver signals to adaptive immune cells and contribute to their activation, one important function of DCs is to present antigens in the form of major histocompatibility complex (MHC) class II. Lysosomal degradation is essential to pathogen degradation and antigen presentation. Intriguingly, the autophagy flux contains the formation of lysosomes, and in some cases, the autophagosomes fused with lysosomes are conducted by autophagy and are also used to degrade pathogens. Thus, autophagy in DCs can serve an effective role to trigger the adaptive immune system (Germic et al., 2019). Upregulation of ATG5 promotes the expression of CD36 and enhances MHC-II antigen presentation in DCs, which demonstrates that autophagy positively results in DC activity (Oh and Lee 2019). In the LPS-induced injury model, the researchers observed enhanced autophagy in DCs. The mechanism of this autophagy may be caused by local hypoxia to upregulate the phosphorylation levels of AKT, ERK, P38, and NF-κB, thereby activating DCs with inflammatory factors such as IL-1β, IL-18, and TNF-α release (Monaci et al., 2020). These inflammatory factors are bound to aggravate liver fibrosis. From previous studies, we find that at least three distinct types of canonical autophagy coexist in dendritic cells, including microautophagy, which involves lysosomal capturing of cytoplasmic components through various modifications on its membrane; chaperone-mediated induced autophagy; and macroautophagy (Ghislat and Lawrence 2018). Even though limited evidence has clarified the function of DC autophagy in liver fibrosis, it can be speculated that excessive autophagy in DCs promotes their activation to remodel the hepatic immune environment and ultimately cause HSC activation. Therefore, proper inhibition of autophagy in hepatic DCs will be beneficial to the prevention and treatment of liver fibrosis.

### 3.4 Natural Killer Cells

Compared with DCs, the proportion of NKs in hepatocytes is much higher than that in peripheral blood, with NKs accounting for nearly 50% of lymphocytes (Abel et al., 2018). Similar to the literal description, the biological function of NKs is in attacking pathogens to maintain homeostasis. NKs are cytotoxic and have effector functions with cytokines, and the ability to be cytotoxic makes it important for these cells to be able to distinguish between target cells and healthy “self” cells. NK cells have a variety of activated and inhibited cell surface receptors that regulate NK cell activity (Vivier et al., 2008). NKs exert potent function in antifibrosis in two different ways. On the one hand, NKs attack and kill HSCs directly in the early stage of HSC activation, but if the HSCs were in the quiescent condition or fully activated, the NKs do not function. On the other hand, NKs release IFN-γ to induce the apoptosis of HSCs (Luci et al., 2019). Meanwhile, the secreted IFN-γ can upregulate the expression of NKG2D and TRAIL on NKs to enhance the NK activities of killing HSCs in a manner of autocrine function (Glassner et al., 2012). Moreover, the fully activated HSCs can release transforming growth factor-beta (TGF-beta) to impair the killing effect of NKs, which explains the dysfunction of NKs in killing fully activated HSCs (Shi et al., 2017). Hence, the utilization of TGF-beta inhibitors is a possible method to strengthen the capability of NKs in the treatment of liver fibrosis.

Recent experimental evidence has preliminarily illustrated the function of autophagy in NKs. NKs can induce mitophagy in the injury caused by a virus infection and maintain the survival of NKs by clearing the damaged mitochondria inside the cell. In addition, autophagy is essential in the process of NK transition from effector cells to long-life memory cells. Drug induction of autophagy enhances memory formation of NKs through an ATG3-dependent mechanism so that NKs can maintain the ability to eliminate the virus and other pathogenic factors (O'Sullivan et al., 2015). Deletion of ATG5, a vital protein involved in autophagy formation, was proved to cause progressive mitochondrial damage and excessive generation of ROS to interrupt the development of NKs and even death (O'Sullivan et al., 2016). In addition, the interaction between ATG7 (another player essential to autophagy) and FOXO1 (forkhead box O1) contributes to the formation of autophagy flux in the cytosol of immature NKs to accelerate the maturation (Huang et al., 2019). These findings demonstrate that proper activation of autophagy is critical to the maturation of NKs, which subsequently enhance the capability of antifibrosis in the liver.

### 3.5 Natural Killer T Cells

Natural killer T cells (NKTs) are innate-like T cell subsets that are abundant in the human liver. NKTs are quite different from the conventional T cells, for they recognize glycolipid antigens presented by the MHC-I molecule, CD1d, which is usually expressed on the cellular membrane of T cells and NKs (Exley et al., 2017). According to the expression type of T cell receptor (TCR), response to glycolipid antigen, and CD1d dependence during development, NKTs can be divided into three types: type I, type II, and type III NKTs. Type I NKTs, also known as invariant NKT cells (iNKTs), express the constant TCRα chain, co-expressed the Vβ chain, and are activated by the *α* galactose amide delivered by the CD1d molecule. iNKTs account for 95% of the total number of lymphocytes in the liver. The narrow definition of NKTs refers to iNKT cells (Wang and Yin 2015), which are also the cell types that we summarize here. After activation, the NKTs release proinflammatory cytokines and interact with other types of innate immune cells (Nilsson et al., 2020). NKTs can not only kill target cells directly but also produce a variety of cytokines, thus playing an important role in liver injury, liver fibrosis, liver regeneration, and the occurrence and development of liver cancer (Gao et al., 2009). Similar to NKs, the activation of NKTs also exerts an antifibrosis role by directly killing partially activated HSCs and rapidly secreting IFN-γ to induce apoptosis of HSCs (Ravichandran et al., 2019). Activated HSCs usually have high expression of NKG2D (natural killer group 2 member D) ligand Rae1(retinoic acid early inducible 1), IL-30 stimulates the high expression of NKG2D in NKTs, and NKTs eventually kill the active HSCs and improve liver fibrosis by combining nKG2D-RAE1 with highly specific targeted activated HSCs (Mitra et al., 2014).

Previous studies have suggested that autophagy is deeply involved in the development and maturation of NKTs. By deleting the expression of ATG7, an important gene in autophagy flux formation, the thymic NKT development is blocked at the very early stage, which is different from conventional T cells. Meanwhile, during the NKT differentiation, the phenomena of autophagy significantly increase (Salio et al., 2014). If autophagy was blocked by deleting ATG5 or ATG7, the number of NKTs is significantly reduced, and the function of NKTs is disrupted (Pei et al., 2015).

The function of autophagy in NKTs, which is also involved in the progression of liver fibrosis, is lacking investigation. However, we can assume that the proper existence of autophagy in NKTs will promote their maturation and facilitate the function of killing HSCs.

## 4 Conclusion and Perspective

To date, emerging evidence has proven that innate immunity plays an important role in liver fibrosis. Activation of HSCs and the remodeling of the hepatic microenvironment by the accumulation of the ECM are essential to the initiation and progression of liver fibrosis. The innate immune cells, such as Kupffer cells, NK cells, NKT cells, and neutrophils, are essential to prevent fibrosis by either directly attacking HSCs or releasing cytokines to facilitate the apoptosis of HSCs, consequently maintaining homeostasis in the liver (Table 1). However, the M2 type of Kupffer cells and DCs generally contribute to the progression of liver fibrosis. For these innate immune cells, the proper activation of autophagy is beneficial to their maturation and biological function, such as the production of cytokines.

**TABLE 1:** Relationship between major innate immune cells and autophagy in liver fibrosis.

Cell type	Interaction with autophagy	Role in fibrosis	References
Kupffer cells	KC autophagy limits IL1A and IL1B secretion	Reduce HSC activation to a certain extent and limit the progression of liver fibrosis	Cai et al. (2018); Lodder et al. (2015)
Neutrophils	Neutrophil autophagy increases sensitivity of damaging factors	Increase neutrophil scavenging or damaging factors and attenuates progression of liver fibrosis	Calvente et al. (2019); Park et al. (2017)
DCs	Autophagy positivity results in DC activity	Hyperactivation of DCs may be a contributing factor to the inflammatory microenvironment of HSCs, further leading to fibrosis progression	Oh and Lee, (2019); Blois et al. (2014)
NKs	Proper activation of autophagy is critical to maturation of NKs	NKs exert potent function in antifibrosis	Luci et al. (2019); Huang et al. (2019)
NKTs	Blocking autophagy causes NKT function disruption	Activation of NKTs can directly kill partially activated HSCs and rapidly secreting IFN-y to induce apoptosis of HSCs	Ravichandran et al. (2019); Pei et al. (2015)

Due to the complication of innate immune cell types and their functions in fibrosis, the function of autophagy depends on innate immune cells. The functions of autophagy are various in cells. For most of the immune cells, the proper extent of autophagy can promote their development and maturation, while excessive autophagy will cause a sharp decrease. Thus, for some innate cell types, such as M1 type of Kupffer cells, NK cells, NKT cells, and neutrophils, the appropriate degree of autophagy can promote their development and maturation, which finally exert the function of antifibrosis; however, the pathological autophagy of these cells can enhance the progression of liver fibrosis. Nevertheless, the agents that trigger autophagy in the M2 type of Kupffer cells and DCs are beneficial to patients suffering from liver fibrosis.

Given the complicated conditions of innate immune cells and autophagy, the therapeutic strategy which targets autophagy in innate immune cells is difficult. In the future, accurate determination of innate cell types in the liver is important before using drugs to stimulate or inhibit autophagy. Meanwhile, immunomodulators in clinical operations should be taken into consideration in the therapy of liver fibrosis.

In summary, the observations in the review strongly support the conclusion that autophagy is crucial for the morphological and biological function of the innate immune system in the liver to prevent the initiation and progression of hepatic fibrosis.

## References

[B1] Abdullah Z. Knolle P. A. (2017). Liver Macrophages in Healthy and Diseased Liver. Pflugers Arch. 469, 553–560. 10.1007/s00424-017-1954-6 28293730

[B2] Abel A. M. Yang C. Thakar M. S. Malarkannan S. (2018). Natural Killer Cells: Development, Maturation, and Clinical Utilization. Front. Immunol. 9, 1869. 10.3389/fimmu.2018.01869 30150991 PMC6099181

[B3] Affo S. Yu L. X. Schwabe R. F. (2017). The Role of Cancer-Associated Fibroblasts and Fibrosis in Liver Cancer. Annu. Rev. Pathol. 12, 153–186. 10.1146/annurev-pathol-052016-100322 27959632 PMC5720358

[B4] Allaire M. Rautou P. E. Codogno P. Lotersztajn S. (2019). Autophagy in Liver Diseases: Time for Translation? J. Hepatol. 70, 985–998. 10.1016/j.jhep.2019.01.026 30711404

[B5] Ambade A. Lowe P. Kodys K. Catalano D. Gyongyosi B. Cho Y. (2019). Pharmacological Inhibition of CCR2/5 Signaling Prevents and Reverses Alcohol-Induced Liver Damage, Steatosis, and Inflammation in Mice. Hepatology 69, 1105–1121. 10.1002/hep.30249 30179264 PMC6393202

[B6] Arsent'eva N. A. Semenov A. V. Lyubimova N. E. Ostankov Y. V. Elezo D. S. Kudryavtsev I. V. (2015). Chemokine Receptors CXCR3 and CCR6 and Their Ligands in the Liver and Blood of Patients with Chronic Hepatitis C. Bull. Exp. Biol. Med. 160, 252–255. 10.1007/s10517-015-3142-z 26631389

[B7] Baglieri J. Brenner D. A. Kisseleva T. (2019). The Role of Fibrosis and Liver-Associated Fibroblasts in the Pathogenesis of Hepatocellular Carcinoma. Int. J. Mol. Sci. 20, 1723. 10.3390/ijms20071723 PMC6479943 30959975

[B8] Bai F. Huang Q. Wei J. Lv S. Chen Y. Liang C. (2018). Gypsophila elegans Isoorientin-2″-O-α-L-Arabinopyranosyl Ameliorates Porcine Serum-Induced Immune Liver Fibrosis by Inhibiting NF-Κb Signaling Pathway and Suppressing HSC Activation. Int. Immunopharmacology 54, 60–67. 10.1016/j.intimp.2017.10.028 29107862

[B9] Blois S. M. Piccioni F. Freitag N. Tirado-González I. Moschansky P. Lloyd R. (2014). Dendritic Cells Regulate Angiogenesis Associated with Liver Fibrogenesis. Angiogenesis 17, 119–128. 10.1007/s10456-013-9382-5 24068342

[B10] Borkham-Kamphorst E. Weiskirchen R. (2016). The PDGF System and its Antagonists in Liver Fibrosis. Cytokine Growth Factor. Rev. 28, 53–61. 10.1016/j.cytogfr.2015.10.002 26547628

[B11] Bruns I. Lucas D. Pinho S. Ahmed J. Lambert M. P. Kunisaki Y. (2014). Megakaryocytes Regulate Hematopoietic Stem Cell Quiescence through CXCL4 Secretion. Nat. Med. 20, 1315–1320. 10.1038/nm.3707 25326802 PMC4258871

[B12] Cai X. Li Z. Zhang Q. Qu Y. Xu M. Wan X. (2018). CXCL6-EGFR-induced Kupffer Cells Secrete TGF-Β1 Promoting Hepatic Stellate Cell Activation via the SMAD2/BRD4/C-MYC/EZH2 Pathway in Liver Fibrosis. J. Cel Mol Med 22, 5050–5061. 10.1111/jcmm.13787 PMC6156397 30106235

[B13] Calvente C. J. Tameda M. Johnson C. D. Del Pilar H. Lin Y. C. Adronikou N. (2019). Neutrophils Contribute to Spontaneous Resolution of Liver Inflammation and Fibrosis via microRNA-223. J. Clin. Invest. 129, 4091–4109. 10.1172/JCI122258 31295147 PMC6763256

[B14] Chen Y. Tian Z. (2020). Roles of Hepatic Innate and Innate-like Lymphocytes in Nonalcoholic Steatohepatitis. Front. Immunol. 11, 1500. 10.3389/fimmu.2020.01500 32765518 PMC7378363

[B15] Chen Z. Jain A. Liu H. Zhao Z. Cheng K. (2019). Targeted Drug Delivery to Hepatic Stellate Cells for the Treatment of Liver Fibrosis. J. Pharmacol. Exp. Ther. 370, 695–702. 10.1124/jpet.118.256156 30886124 PMC6806344

[B16] Cheng D. Chai J. Wang H. Fu L. Peng S. Ni X. (2021). Hepatic Macrophages: Key Players in the Development and Progression of Liver Fibrosis. Liver Int. 41, 2279–2294. 10.1111/liv.14940 33966318

[B17] Cosin-Roger J. Simmen S. Melhem H. Atrott K. Frey-Wagner I. Hausmann M. (2017). Hypoxia Ameliorates Intestinal Inflammation through NLRP3/mTOR Downregulation and Autophagy Activation. Nat. Commun. 8, 98. 10.1038/s41467-017-00213-3 28740109 PMC5524634

[B18] Deretic V. Levine B. (2018). Autophagy Balances Inflammation in Innate Immunity. Autophagy 14, 243–251. 10.1080/15548627.2017.1402992 29165043 PMC5902214

[B19] Dewidar B. Meyer C. Dooley S. Meindl-Beinker A. N. (2019). TGF-beta in Hepatic Stellate Cell Activation and Liver Fibrogenesis-Updated. Cells, 8, 1419. 10.3390/cells8111419 PMC6912224 31718044

[B20] Exley M. A. Wilson S. B. Balk S. P. (2017). Isolation and Functional Use of Human NKT Cells. Curr. Protoc. Immunol. 119, 14–20. 10.1002/cpim.33 29091262

[B21] Fabre T. Molina M. F. Soucy G. Goulet J. P. Willems B. Villeneuve J. P. (2018). Type 3 Cytokines IL-17A and IL-22 Drive TGF-β-dependent Liver Fibrosis. Sci. Immunol. 3, eaar7754. 10.1126/sciimmunol.aar7754 30366940

[B22] Feng J. Wang C. Liu T. Li J. Wu L. Yu Q. (2019). Procyanidin B2 Inhibits the Activation of Hepatic Stellate Cells and Angiogenesis via the Hedgehog Pathway during Liver Fibrosis. J. Cel Mol Med 23, 6479–6493. 10.1111/jcmm.14543 PMC6714206 31328391

[B23] Gan J. Zheng S. J. Mao X. R. Li J. F. (2021). Invariant Natural Killer T Cells: Not to Be Ignored in Liver Disease. J. Dig. Dis. 22, 136–142. 10.1111/1751-2980.12968 33421264

[B24] Gao B. Radaeva S. Park O. (2009). Liver Natural Killer and Natural Killer T Cells: Immunobiology and Emerging Roles in Liver Diseases. J. Leukoc. Biol. 86, 513–528. 10.1189/JLB.0309135 19542050 PMC2735282

[B25] Gao J. Wei B. De Assuncao T. M. Liu Z. Hu X. Ibrahim S. (2020). Hepatic Stellate Cell Autophagy Inhibits Extracellular Vesicle Release to Attenuate Liver Fibrosis. J. Hepatol. 73, 1144–1154. 10.1016/j.jhep.2020.04.044 32389810 PMC7572579

[B26] Ge J. Yan Q. Wang Y. Cheng X. Song D. Wu C. (2020). IL-10 Delays the Degeneration of Intervertebral Discs by Suppressing the P38 MAPK Signaling Pathway. Free Radic. Biol. Med. 147, 262–270. 10.1016/j.freeradbiomed.2019.12.040 31883468

[B27] Germic N. Frangez Z. Yousefi S. Simon H. U. (2019). Regulation of the Innate Immune System by Autophagy: Monocytes, Macrophages, Dendritic Cells and Antigen Presentation. Cell Death Differ 26, 715–727. 10.1038/s41418-019-0297-6 30737475 PMC6460400

[B28] Ghislat G. Lawrence T. (2018). Autophagy in Dendritic Cells. Cell Mol Immunol 15, 944–952. 10.1038/cmi.2018.2 29578531 PMC6207777

[B29] Glässner A. Eisenhardt M. Krämer B. Körner C. Coenen M. Sauerbruch T. (2012). NK Cells from HCV-Infected Patients Effectively Induce Apoptosis of Activated Primary Human Hepatic Stellate Cells in a TRAIL-, FasL- and NKG2D-dependent Manner. Lab. Invest. 92, 967–977. 10.1038/labinvest.2012.54 22449797

[B30] Hammoutene A. Biquard L. Lasselin J. Kheloufi M. Tanguy M. Vion A. C. (2020). A Defect in Endothelial Autophagy Occurs in Patients with Non-alcoholic Steatohepatitis and Promotes Inflammation and Fibrosis. J. Hepatol. 72, 528–538. 10.1016/j.jhep.2019.10.028 31726115

[B31] Higashi T. Friedman S. L. Hoshida Y. (2017). Hepatic Stellate Cells as Key Target in Liver Fibrosis. Adv. Drug Deliv. Rev. 121, 27–42. 10.1016/j.addr.2017.05.007 28506744 PMC5682243

[B32] Hossain M. Kubes P. (2019). Innate Immune Cells Orchestrate the Repair of Sterile Injury in the Liver and beyond. Eur. J. Immunol. 49, 831–841. 10.1002/eji.201847485 31001813

[B33] Hou L. S. Zhang Y. W. Li H. Wang W. Huan M. L. Zhou S. Y. (2022). The Regulatory Role and Mechanism of Autophagy in Energy Metabolism-Related Hepatic Fibrosis. Pharmacol. Ther. 234, 108117. 10.1016/j.pharmthera.2022.108117 35077761

[B34] Hu Y. X. Han X. S. Jing Q. (2019). Autophagy in Development and Differentiation. Adv. Exp. Med. Biol. 1206, 469–487. 10.1007/978-981-15-0602-4_22 31776999

[B35] Huang P. Wang F. Yang Y. Lai W. Meng M. Wu S. (2019). Hematopoietic-Specific Deletion of Foxo1 Promotes NK Cell Specification and Proliferation. Front. Immunol. 10, 1016. 10.3389/fimmu.2019.01016 31139183 PMC6519137

[B36] Huang R. Liu W. (2015). Identifying an Essential Role of Nuclear LC3 for Autophagy. Autophagy 11, 852–853. 10.1080/15548627.2015.1038016 25945743 PMC4509442

[B37] Izawa T. Murakami H. Wijesundera K. K. Golbar H. M. Kuwamura M. Yamate J. (2014). Inflammatory Regulation of Iron Metabolism during Thioacetamide-Induced Acute Liver Injury in Rats. Exp. Toxicol. Pathol. 66, 155–162. 10.1016/j.etp.2013.12.002 24373749

[B38] Jenne C. N. Kubes P. (2013). Immune Surveillance by the Liver. Nat. Immunol. 14, 996–1006. 10.1038/ni.2691 24048121

[B39] Ji G. Ma L. Yao H. Ma S. Si X. Wang Y. (2020). Precise Delivery of Obeticholic Acid via Nanoapproach for Triggering Natural Killer T Cell-Mediated Liver Cancer Immunotherapy. Acta Pharm. Sin B 10, 2171–2182. 10.1016/j.apsb.2020.09.004 33304784 PMC7715527

[B40] Jiang G. M. Tan Y. Wang H. Peng L. Chen H. T. Meng X. J. (2019). The Relationship between Autophagy and the Immune System and its Applications for Tumor Immunotherapy. Mol. Cancer 18, 17. 10.1186/s12943-019-0944-z 30678689 PMC6345046

[B41] Kakino S. Ohki T. Nakayama H. Yuan X. Otabe S. Hashinaga T. (2018). Pivotal Role of TNF-α in the Development and Progression of Nonalcoholic Fatty Liver Disease in a Murine Model. Horm. Metab. Res. 50, 80–87. 10.1055/s-0043-118666 28922680

[B42] Karsdal M. A. Nielsen S. H. Leeming D. J. Langholm L. L. Nielsen M. J. Manon-Jensen T. (2017). The Good and the Bad Collagens of Fibrosis - Their Role in Signaling and Organ Function. Adv. Drug Deliv. Rev. 121, 43–56. 10.1016/j.addr.2017.07.014 28736303

[B43] Katsuragi Y. Ichimura Y. Komatsu M. (2015). p62/SQSTM1 Functions as a Signaling Hub and an Autophagy Adaptor. FEBS J. 282, 4672–4678. 10.1111/febs.13540 26432171

[B44] Li W. Zhang L. (2019). Regulation of ATG and Autophagy Initiation. Adv. Exp. Med. Biol. 1206, 41–65. 10.1007/978-981-15-0602-4_2 31776979

[B45] Li X. Liu R. Wang Y. Zhu W. Zhao D. Wang X. (2020a). Cholangiocyte-Derived Exosomal lncRNA H19 Promotes Macrophage Activation and Hepatic Inflammation under Cholestatic Conditions. Cells 9, 190. 10.3390/cells9010190 PMC7016679 31940841

[B46] Li Y. Liu R. Wu J. Li X. (2020b). Self-eating: Friend or Foe? the Emerging Role of Autophagy in Fibrotic Diseases. Theranostics 10, 7993–8017. 10.7150/thno.47826 32724454 PMC7381749

[B47] Liu N. Feng J. Lu X. Yao Z. Liu Q. Lv Y. (2019). Isorhamnetin Inhibits Liver Fibrosis by Reducing Autophagy and Inhibiting Extracellular Matrix Formation via the TGF-β1/Smad3 and TGF-Β1/p38 MAPK Pathways. Mediators Inflamm. 2019, 6175091. 10.1155/2019/6175091 31467486 PMC6701280

[B48] Liu Y. M. Cong S. Cheng Z. Hu Y. X. Lei Y. Zhu L. L. (2020). Platycodin D Alleviates Liver Fibrosis and Activation of Hepatic Stellate Cells by Regulating JNK/c-JUN Signal Pathway. Eur. J. Pharmacol. 876, 172946. 10.1016/j.ejphar.2020.172946 31996320

[B49] Lodder J. Denaës T. Chobert M. N. Wan J. El-Benna J. Pawlotsky J. M. (2015). Macrophage Autophagy Protects against Liver Fibrosis in Mice. Autophagy 11, 1280–1292. 10.1080/15548627.2015.1058473 26061908 PMC4590651

[B50] Lorincz P. Juhasz G. (2020). Autophagosome-Lysosome Fusion. J. Mol. Biol. 432, 2462–2482. 10.1016/j.jmb.2019.10.028 31682838

[B51] Luci C. Vieira E. Perchet T. Gual P. Golub R. (2019). Natural Killer Cells and Type 1 Innate Lymphoid Cells Are New Actors in Non-alcoholic Fatty Liver Disease. Front. Immunol. 10, 1192. 10.3389/fimmu.2019.01192 31191550 PMC6546848

[B52] Luedde T. Schwabe R. F. (2011). NF-κB in the Liver-Llinking Injury, Fibrosis and Hepatocellular Carcinoma. Nat. Rev. Gastroenterol. Hepatol. 8, 108–118. 10.1038/nrgastro.2010.213 21293511 PMC3295539

[B53] Madhavi Y. V. Gaikwad N. Yerra V. G. Kalvala A. K. Nanduri S. Kumar A. (2019). Targeting AMPK in Diabetes and Diabetic Complications: Energy Homeostasis, Autophagy and Mitochondrial Health. Curr. Med. Chem. 26, 5207–5229. 10.2174/0929867325666180406120051 29623826

[B54] Matsuda M. Tsurusaki S. Miyata N. Saijou E. Okochi H. Miyajima A. (2018). Oncostatin M Causes Liver Fibrosis by Regulating Cooperation between Hepatic Stellate Cells and Macrophages in Mice. Hepatology 67, 296–312. 10.1002/hep.29421 28779552

[B55] Meng D. Li Z. Wang G. Ling L. Wu Y. Zhang C. (2018). Carvedilol Attenuates Liver Fibrosis by Suppressing Autophagy and Promoting Apoptosis in Hepatic Stellate Cells. Biomed. Pharmacother. 108, 1617–1627. 10.1016/j.biopha.2018.10.005 30372864

[B56] Miao Q. Bian Z. Tang R. Zhang H. Wang Q. Huang S. (2015). Emperipolesis Mediated by CD8 T Cells Is a Characteristic Histopathologic Feature of Autoimmune Hepatitis. Clin. Rev. Allergy Immunol. 48, 226–235. 10.1007/s12016-014-8432-0 25051956

[B57] Mitra A. Satelli A. Yan J. Xueqing X. Gagea M. Hunter C. A. (2014). IL-30 (IL27p28) Attenuates Liver Fibrosis through Inducing NKG2D-Rae1 Interaction between NKT and Activated Hepatic Stellate Cells in Mice. Hepatology 60, 2027–2039. 10.1002/hep.27392 25351459 PMC4245364

[B58] Mizushima N. (2010). The Role of the Atg1/ULK1 Complex in Autophagy Regulation. Curr. Opin. Cel Biol 22, 132–139. 10.1016/j.ceb.2009.12.004 20056399

[B59] Monaci S. Aldinucci C. Rossi D. Giuntini G. Filippi I. Ulivieri C. (2020). Hypoxia Shapes Autophagy in LPS-Activated Dendritic Cells. Front. Immunol. 11, 573646. 10.3389/fimmu.2020.573646 33329536 PMC7734254

[B60] Moon A. M. Singal A. G. Tapper E. B. (2020). Contemporary Epidemiology of Chronic Liver Disease and Cirrhosis. Clin. Gastroenterol. Hepatol. 18, 2650–2666. 10.1016/j.cgh.2019.07.060 31401364 PMC7007353

[B61] Mowers E. E. Sharifi M. N. Macleod K. F. (2018). Functions of Autophagy in the Tumor Microenvironment and Cancer Metastasis. FEBS J. 285, 1751–1766. 10.1111/febs.14388 29356327 PMC5992019

[B62] Mridha A. R. Wree A. Robertson A. A. B. Yeh M. M. Johnson C. D. Van Rooyen D. M. (2017). NLRP3 Inflammasome Blockade Reduces Liver Inflammation and Fibrosis in Experimental NASH in Mice. J. Hepatol. 66, 1037–1046. 10.1016/j.jhep.2017.01.022 28167322 PMC6536116

[B63] Niehaus C. E. Strunz B. Cornillet M. Falk C. S. Schnieders A. Maasoumy B. (2020). MAIT Cells Are Enriched and Highly Functional in Ascites of Patients with Decompensated Liver Cirrhosis. Hepatology 72, 1378–1393. 10.1002/hep.31153 32012321

[B64] Nilsson J. Hörnberg M. Schmidt-Christensen A. Linde K. Nilsson M. Carlus M. (2020). NKT Cells Promote Both Type 1 and Type 2 Inflammatory Responses in a Mouse Model of Liver Fibrosis. Sci. Rep. 10, 21778. 10.1038/s41598-020-78688-2 33311540 PMC7732838

[B65] O'sullivan T. E. Geary C. D. Weizman O. E. Geiger T. L. Rapp M. Dorn G. W. 2nd (2016). Atg5 Is Essential for the Development and Survival of Innate Lymphocytes. Cell Rep 15, 1910–1919. 10.1016/j.celrep.2016.04.082 27210760 PMC4889506

[B66] O'sullivan T. E. Johnson L. R. Kang H. H. Sun J. C. (2015). BNIP3- and BNIP3L-Mediated Mitophagy Promotes the Generation of Natural Killer Cell Memory. Immunity 43, 331–342. 10.1016/j.immuni.2015.07.012 26253785 PMC5737626

[B67] Oh D. S. Lee H. K. (2019). Autophagy Protein ATG5 Regulates CD36 Expression and Anti-tumor MHC Class II Antigen Presentation in Dendritic Cells. Autophagy 15, 2091–2106. 10.1080/15548627.2019.1596493 30900506 PMC6844530

[B68] Pang Y. Kartsonaki C. Turnbull I. Guo Y. Clarke R. Chen Y. (2018). Diabetes, Plasma Glucose, and Incidence of Fatty Liver, Cirrhosis, and Liver Cancer: A Prospective Study of 0.5 Million People. Hepatology 68, 1308–1318. 10.1002/hep.30083 29734463 PMC6220764

[B69] Papayannopoulos V. (2018). Neutrophil Extracellular Traps in Immunity and Disease. Nat. Rev. Immunol. 18, 134–147. 10.1038/nri.2017.105 28990587

[B70] Park S. Y. Shrestha S. Youn Y. J. Kim J. K. Kim S. Y. Kim H. J. (2017). Autophagy Primes Neutrophils for Neutrophil Extracellular Trap Formation during Sepsis. Am. J. Respir. Crit. Care Med. 196, 577–589. 10.1164/rccm.201603-0596OC 28358992

[B71] Parola M. Pinzani M. (2019). Liver Fibrosis: Pathophysiology, Pathogenetic Targets and Clinical Issues. Mol. Aspects Med. 65, 37–55. 10.1016/j.mam.2018.09.002 30213667

[B72] Pei B. Zhao M. Miller B. C. Véla J. L. Bruinsma M. W. Virgin H. W. (2015). Invariant NKT Cells Require Autophagy to Coordinate Proliferation and Survival Signals during Differentiation. J. Immunol. 194, 5872–5884. 10.4049/jimmunol.1402154 25926673 PMC4458460

[B73] Peng Y. Li Y. He Y. Wei Q. Xie Q. Zhang L. (2018). The Role of Neutrophil to Lymphocyte Ratio for the Assessment of Liver Fibrosis and Cirrhosis: a Systematic Review. Expert Rev. Gastroenterol. Hepatol. 12, 503–513. 10.1080/17474124.2018.1463158 29629626

[B74] Plaza-Zabala A. Sierra-Torre V. Sierra A. (2017). Autophagy and Microglia: Novel Partners in Neurodegeneration and Aging. Int. J. Mol. Sci. 18, 598. 10.3390/ijms18030598 PMC5372614 28282924

[B75] Pokora Rodak A. Kiciak S. Tomasiewicz K. (2018). Neutrophil-lymphocyte Ratio and Mean Platelet Volume as Predictive Factors for Liver Fibrosis and Steatosis in Patients with Chronic Hepatitis B. Ann. Agric. Environ. Med. 25, 690–692. 10.26444/aaem/99583 30586966

[B76] Pradere J. P. Kluwe J. De Minicis S. Jiao J. J. Gwak G. Y. Dapito D. H. (2013). Hepatic Macrophages but Not Dendritic Cells Contribute to Liver Fibrosis by Promoting the Survival of Activated Hepatic Stellate Cells in Mice. Hepatology 58, 1461–1473. 10.1002/hep.26429 23553591 PMC3848418

[B77] Qu Y. Zhang Q. Cai X. Li F. Ma Z. Xu M. (2017). Exosomes Derived from miR-181-5p-Modified Adipose-Derived Mesenchymal Stem Cells Prevent Liver Fibrosis via Autophagy Activation. J. Cel Mol Med 21, 2491–2502. 10.1111/jcmm.13170 PMC5618698 28382720

[B78] Ravichandran G. Neumann K. Berkhout L. K. Weidemann S. Langeneckert A. E. Schwinge D. (2019). Interferon-γ-dependent Immune Responses Contribute to the Pathogenesis of Sclerosing Cholangitis in Mice. J. Hepatol. 71, 773–782. 10.1016/j.jhep.2019.05.023 31173810

[B79] Riffelmacher T. Clarke A. Richter F. C. Stranks A. Pandey S. Danielli S. (2017). Autophagy-Dependent Generation of Free Fatty Acids Is Critical for Normal Neutrophil Differentiation. Immunity 47, 466–e5. 10.1016/j.immuni.2017.08.005 28916263 PMC5610174

[B80] Roh Y. S. Seki E. (2018). Chemokines and Chemokine Receptors in the Development of NAFLD. Adv. Exp. Med. Biol. 1061, 45–53. 10.1007/978-981-10-8684-7_4 29956205

[B81] Sakai Y. Chen G. Ni Y. Zhuge F. Xu L. Nagata N. (2020). DPP-4 Inhibition with Anagliptin Reduces Lipotoxicity-Induced Insulin Resistance and Steatohepatitis in Male Mice. Endocrinology 161, bqaa139. 10.1210/endocr/bqaa139 32790863

[B82] Salio M. Puleston D. J. Mathan T. S. Shepherd D. Stranks A. J. Adamopoulou E. (2014). Essential Role for Autophagy during Invariant NKT Cell Development. Proc. Natl. Acad. Sci. U S A. 111, E5678–E5687. 10.1073/pnas.1413935112 25512546 PMC4284579

[B83] Schnieder J. Mamazhakypov A. Birnhuber A. Wilhelm J. Kwapiszewska G. Ruppert C. (2020). Loss of LRP1 Promotes Acquisition of Contractile-Myofibroblast Phenotype and Release of Active TGF-Β1 from ECM Stores. Matrix Biol. 88, 69–88. 10.1016/j.matbio.2019.12.001 31841706

[B84] Schumacher J. D. Kong B. Wu J. Rizzolo D. Armstrong L. E. Chow M. D. (2020). Direct and Indirect Effects of Fibroblast Growth Factor (FGF) 15 and FGF19 on Liver Fibrosis Development. Hepatology 71, 670–685. 10.1002/hep.30810 31206730 PMC6918008

[B85] Seki E. De Minicis S. Gwak G. Y. Kluwe J. Inokuchi S. Bursill C. A. (2009). CCR1 and CCR5 Promote Hepatic Fibrosis in Mice. J. Clin. Invest. 119, 1858–1870. 10.1172/jci37444 19603542 PMC2701864

[B86] Sepulveda-Crespo D. Resino S. Martinez I. (2021). Strategies Targeting the Innate Immune Response for the Treatment of Hepatitis C Virus-Associated Liver Fibrosis. Drugs 81, 419–443. 10.1007/s40265-020-01458-x 33400242

[B87] Shi J. Zhao J. Zhang X. Cheng Y. Hu J. Li Y. (2017). Activated Hepatic Stellate Cells Impair NK Cell Anti-fibrosis Capacity through a TGF-β-dependent Emperipolesis in HBV Cirrhotic Patients. Sci. Rep. 7, 44544. 10.1038/srep44544 28291251 PMC5349579

[B88] Soysa R. Wu X. Crispe I. N. (2017). Dendritic Cells in Hepatitis and Liver Transplantation. Liver Transpl. 23, 1433–1439. 10.1002/lt.24833 28752938

[B89] Su S. B. Qin S. Y. Xian X. L. Huang F. F. Huang Q. L. Zhangdi H. J. (2021). Interleukin-22 Regulating Kupffer Cell Polarization through STAT3/Erk/Akt Crosstalk Pathways to Extenuate Liver Fibrosis. Life Sci. 264, 118677. 10.1016/j.lfs.2020.118677 33129875

[B90] Sun K. Xu L. Jing Y. Han Z. Chen X. Cai C. (2017). Autophagy-deficient Kupffer Cells Promote Tumorigenesis by Enhancing mtROS-NF-κB-IL1α/β-dependent Inflammation and Fibrosis during the Preneoplastic Stage of Hepatocarcinogenesis. Cancer Lett. 388, 198–207. 10.1016/j.canlet.2016.12.004 28011320

[B91] Théry C. Amigorena S. (2001). The Cell Biology of Antigen Presentation in Dendritic Cells. Curr. Opin. Immunol. 13, 45–51. 10.1016/s0952-7915(00)00180-1 11154916

[B92] Vivier E. Tomasello E. Baratin M. Walzer T. Ugolini S. (2008). Functions of Natural Killer Cells. Nat. Immunol. 9, 503–510. 10.1038/ni1582 18425107

[B93] Wang H. Yin S. (2015). Natural Killer T Cells in Liver Injury, Inflammation and Cancer. Expert Rev. Gastroenterol. Hepatol. 9, 1077–1085. 10.1586/17474124.2015.1056738 26068039

[B94] Weiskirchen R. Tacke F. (2014). Cellular and Molecular Functions of Hepatic Stellate Cells in Inflammatory Responses and Liver Immunology. Hepatobiliary Surg. Nutr. 3, 344–363. 10.3978/j.issn.2304-3881.2014.11.03 25568859 PMC4273119

[B95] Wu Y. Huang M. Sun H. Zhou X. Zhou R. Gu G. (2020). Role of Innate Immunity in Pediatric Post-transplant Idiopathic Liver Fibrosis. Front. Immunol. 11, 2111. 10.3389/fimmu.2020.02111 33193293 PMC7642407

[B96] Wu Z. Han M. Chen T. Yan W. Ning Q. (2010). Acute Liver Failure: Mechanisms of Immune-Mediated Liver Injury. Liver Int. 30, 782–794. 10.1111/j.1478-3231.2010.02262.x 20492514

[B97] Xiang H. Zhang J. Lin C. Zhang L. Liu B. Ouyang L. (2020). Targeting Autophagy-Related Protein Kinases for Potential Therapeutic Purpose. Acta Pharm. Sin B 10, 569–581. 10.1016/j.apsb.2019.10.003 32322463 PMC7161711

[B98] Xiang M. Liu T. Tian C. Ma K. Gou J. Huang R. (2022). Kinsenoside Attenuates Liver Fibro-Inflammation by Suppressing Dendritic Cells via the PI3K-AKT-FoxO1 Pathway. Pharmacol. Res. 177, 106092. 10.1016/j.phrs.2022.106092 35066108 PMC8776354

[B99] Xu F. Liu C. Zhou D. Zhang L. (2016). TGF-β/SMAD Pathway and its Regulation in Hepatic Fibrosis. J. Histochem. Cytochem. 64, 157–167. 10.1369/0022155415627681 26747705 PMC4810800

[B100] Yan S. Huda N. Khambu B. Yin X. M. (2017). Relevance of Autophagy to Fatty Liver Diseases and Potential Therapeutic Applications. Amino Acids 49, 1965–1979. 10.1007/s00726-017-2429-y 28478585 PMC5759960

[B101] Yan X. Zhou R. Ma Z. (2019). Autophagy-Cell Survival and Death. Adv. Exp. Med. Biol. 1206, 667–696. 10.1007/978-981-15-0602-4_29 31777006

[B102] Yan Z. Qin C. Zhao C. Fang Z. (2020). Research Progress of Nanomaterial-Mediated Photodynamic Therapy in Tumor Treatment. J. Nanoparticle Res. 22, 12405. 10.1007/s11051-020-05030-2

[B103] Yang W. Tao Y. Wu Y. Zhao X. Ye W. Zhao D. (2019). Neutrophils Promote the Development of Reparative Macrophages Mediated by ROS to Orchestrate Liver Repair. Nat. Commun. 10, 1076. 10.1038/s41467-019-09046-8 30842418 PMC6403250

[B104] Zhang C. Y. Yuan W. G. He P. Lei J. H. Wang C. X. (2016). Liver Fibrosis and Hepatic Stellate Cells: Etiology, Pathological Hallmarks and Therapeutic Targets. World J. Gastroenterol. 22, 10512–10522. 10.3748/wjg.v22.i48.10512 28082803 PMC5192262

[B105] Zhou J. Huang N. Guo Y. Cui S. Ge C. He Q. (2019). Combined Obeticholic Acid and Apoptosis Inhibitor Treatment Alleviates Liver Fibrosis. Acta Pharm. Sin B 9, 526–536. 10.1016/j.apsb.2018.11.004 31193776 PMC6542786

[B106] Zhou S. Gu J. Liu R. Wei S. Wang Q. Shen H. (2018). Spermine Alleviates Acute Liver Injury by Inhibiting Liver-Resident Macrophage Pro-inflammatory Response through ATG5-dependent Autophagy. Front. Immunol. 9, 948. 10.3389/fimmu.2018.00948 29770139 PMC5940752

[B107] Zhu J. Zhang W. Zhang L. Xu L. Chen X. Zhou S. (2018). IL-7 Suppresses Macrophage Autophagy and Promotes Liver Pathology in Schistosoma Japonicum-Infected Mice. J. Cel Mol Med 22, 3353–3363. 10.1111/jcmm.13610 PMC6010884 29566311

